# Cutaneous Angiomyolipoma—A Distinct Entity That Should Be Separated From Classic Angiomyolipoma: Complete Review of Existing Cases and Defining Fundamental Features

**DOI:** 10.2196/40168

**Published:** 2022-09-27

**Authors:** Natalia Gabriela Sanchez, Alfonsina Angelica Ávila Romay, Eduwiges Martínez Luna, Alvaro Lezid Padilla Rodríguez

**Affiliations:** 1 DIGIPATH: Digital Pathology Laboratory Mexico City Mexico; 2 Instituto Tecnológico y de Estudios Superiores De Monterrey Campus Ciudad de México Mexico City Mexico; 3 Clínica Dermatología Integral Medica Sur Mexico City Mexico; 4 Clínica Dermamedics Morelia, Michoacán Mexico; 5 Escuela de Medicina Universidad Panamericana Campus Ciudad de México Mexico City Mexico

**Keywords:** angiomyolipoma, cutaneous angiomyolipoma, cutaneous mesenchymal tumors, HMB-45

## Abstract

Cutaneous angiomyolipoma is a rare mesenchymal tumor that is demographically, clinically, and immunohistochemically distinct from its renal and extrarenal counterparts. We present a case of cutaneous angiomyolipoma in the right retroauricular area of a 35-year-old male patient and provide a broad systematic review of the literature and the largest compilation of cutaneous angiomyolipomas reported to date. According to the findings presented in this review, we conclude that cutaneous angiomyolipoma should be completely separated from renal and extrarenal angiomyolipomas and therefore be considered a distinct entity in the classification of skin tumors.

## Introduction

Cutaneous soft tissue tumors are a heterogeneous group of neoplasms arising from different dermal and subcutaneous tissue components. Benign tumors vastly outnumber sarcomas [1].

Cutaneous angiomyolipoma (hereinafter described as “cutaneous AML”) is a benign tumor composed of varying proportions of thick-walled blood vessels, mature adipose tissue, and smooth muscle cells arranged in bundles, histologically identical to renal and extrarenal angiomyolipoma (hereinafter described as “classic AML”). Cutaneous AML is extremely rare and is not included in the latest 2018 World Health Organization (WHO) classification of skin tumors [1].

A total of 43 cases have been reported in English and Spanish literature to date; we present a new cutaneous AML in a 35-year-old male, which would represent the 44th case. We present the largest compilation of cutaneous AMLs, describe their clinical and morphological features, and contrast them with classic AMLs.

Our findings reveal that although they share similar histopathologic features, classic and cutaneous AML should be considered separate entities owing to their distinct demographic, clinical, and immunohistochemical features. Immunostains for melanocytic markers (such as monoclonal antibody HMB-45) are crucial in differentiating these 2 entities, being positive in classic AML [2-8] and negative in cutaneous AML. These differences allow us to conclude distinct histogeneses and incorporate cutaneous AML into an independent category in skin soft tissue tumors.

## Case Report

### Case Overview

**Figure 1:**
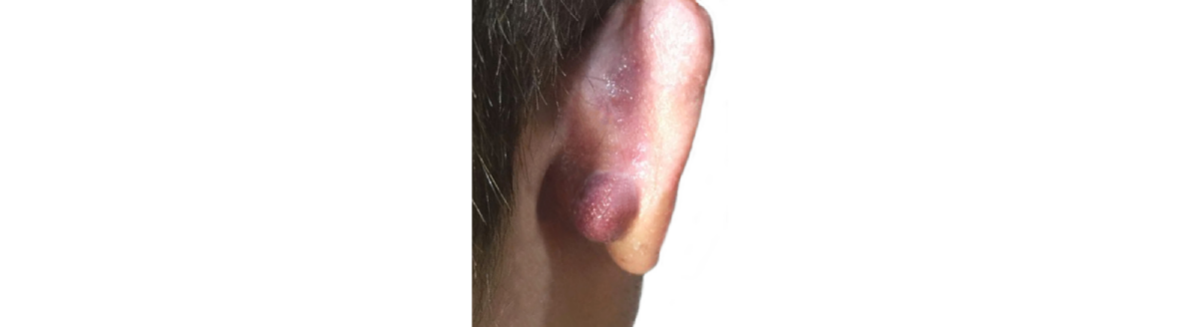
Exophytic nodule localized in the postauricular region of the right ear, adjacent to the earlobe. Erythematous, soft to touch, mobile, measuring 1.7 cm in diameter. Epidermis is intact.

A 35-year-old male patient presented with a mass on his right ear, which progressively increased in size and became painful to touch after local trauma. He was otherwise in good health and had no clinical signs or familiar history of tuberous sclerosis complex (TSC) or classic AML. Physical examination revealed a nodular, erythematous, soft, mobile, subcutaneous mass in the right retroauricular area, which had a diameter of 1.7 cm (Figure 1). Clinical impression suggested a keloid scar versus skin appendage; thus, excision was performed by CO_2_ laser.

### Macroscopic Findings

**Figure 2:**
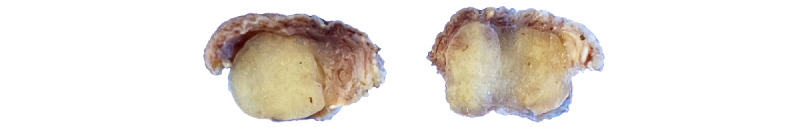
Resected well-circumscribed mass measuring 1.3×0.6 cm with a heterogeneous whitish-yellow appearance.

The excisional skin biopsy showed a subcutaneous nodular mass covered by a rugged grayish-tan epidermal surface. At the cut surface, a well-circumscribed, subepidermal, whitish-yellow, heterogeneous soft mass was present, measuring 1.3 × 0.6 cm (Figure 2).

### Microscopic Features

**Figure 3:**
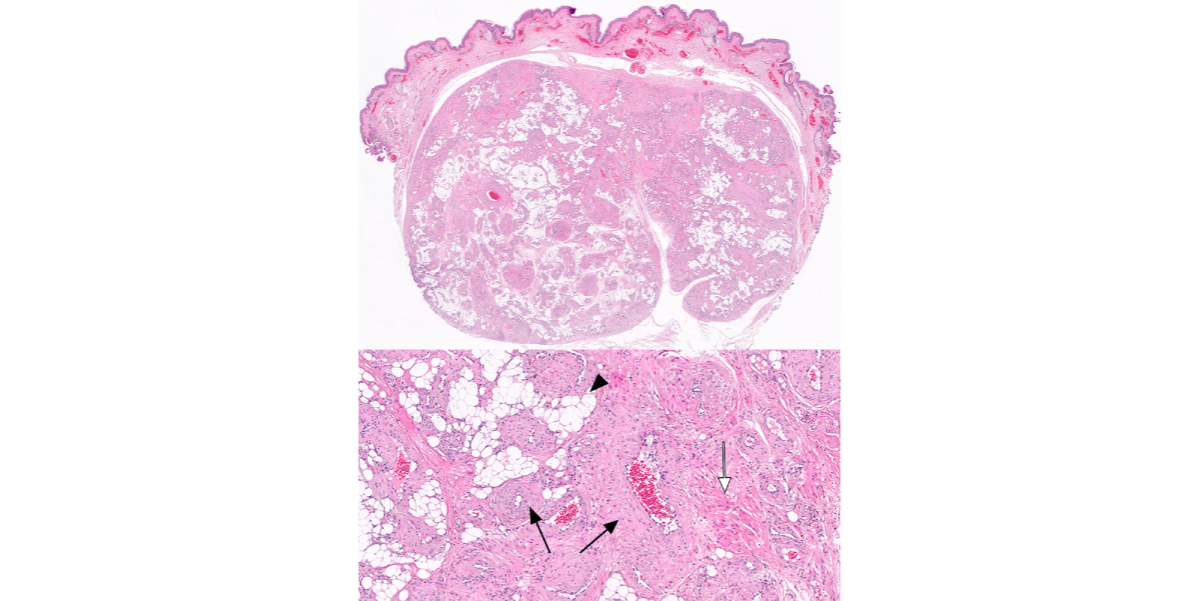
Low-power view demonstrating subcutaneous location and sharply demarcated border of the tumor (hematoxylin-eosin staining, ×10 magnification). The tumor is composed of thick-walled blood vessels (black arrows), mature adipose tissue (arrowhead), and smooth muscle cells arranged in bundles (white arrow; hematoxylin-eosin staining, ×100 magnification).

Hematoxylin-eosin–stained sections revealed a well-circumscribed nodule, a surrounding fibrous pseudocapsule (Figure 3), small or medium blood vessels, adipose tissue, and bundled smooth muscle cells (Figure 3). Cellular pleomorphism, atypia, mitotic figures, and necrosis were absent. The tumor was in the junction between the reticular dermis and the hypodermis. The epidermal surface showed no significant histological changes.

**Figure 4:**
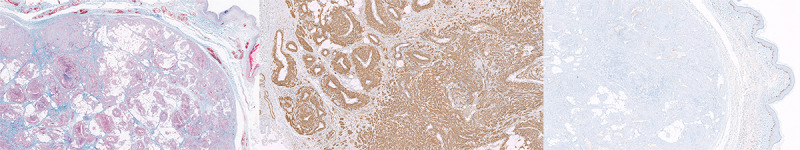
Smooth muscle bundles and vascular smooth muscle stained in red, and fibrous pseudocapsule stained in blue (Masson’s trichrome stain, ×20 magnification). Immunostaining showing the muscular components of the tumor (smooth muscle actin, ×100 magnification). Completely negative immunostaining for melanocytic markers in the tumor, with a positive reaction in the epidermal melanocytes (Melanoma Cocktail: HMB-45, MART-1, and Tyrosinase; ×50 magnification).

Masson’s trichrome staining revealed smooth muscle bundles (red), muscular blood vessels (red), stromal connective tissue (blue), and the fibrous pseudocapsule (blue) (Figure 4).

Immunohistochemical analysis using the Ventana BenchMark ULTRA platform with the UltraView detection system revealed positive staining for smooth muscle actin (SMA, clone 1A4) (Figure 4) and negative staining for the following melanocytic markers: anti-melanosome (monoclonal antibody HMB-45), MART-1 (Melan-A, clone A103) and Tyrosinase (clone T311; Figure 4). Both positive and negative controls were adequate for all studies.

Based on the findings, the case was diagnosed as a completely excised cutaneous AML. The patient had no recurrence at 1 month follow-up.

## Discussion

### Background

Soft tissue cutaneous tumors are a heterogeneous group of neoplasms originating from distinct dermal and subcutaneous tissue components. The most common benign mesenchymal tumors are lipomas, dermatofibromas (fibrous histiocytomas), vascular or smooth muscle lesions, and nerve sheath tumors. These tumors are usually superficial and small, measuring less than 5 cm, and present clinically as painless plaques or nodules with variable growth rates. Benign tumors are generally successfully treated with complete excision and rarely recur locally [1].

Cutaneous AML was first described by Argenyi et al [9] in 1986. Since then, according to a comprehensive review of English and Spanish literature (PubMed, SciELO, and Google Scholar) by searching the databases using the terms *cutaneous angiomyolipoma* and *cutaneous angiolipoleiomyoma* without date restrictions, 43 patients with cutaneous AML have been reported to date (Table 1) [10-39]. To our knowledge, our case is the 44th case of cutaneous AML described.

Data analysis from all reported cases of cutaneous AML reveals significant differences with classic AML and should therefore be classified as separate clinicopathological entities. To support this statement, we first describe classic AML, establish clinical and diagnostic criteria for cutaneous AMLs based on all cases reported to date, and finally contrast its characteristics with those of classic AML.

**Table 1:** Cutaneous angiomyolipoma: clinical and morphological features of all cases published to date.

Author (year)	Case	Sex (age in years)	Clinical diagnosis	Disease evolution time (years)	Location	Symptoms	Size (cm)	Microscopic findings	Melanocytic markers	Treatment	Recurrence
Argenyi et al [10] (1986)	1	Male (67)	Epidermal cyst	40	Right helix	Not specified (NS)	1×1	Adipose tissue (AT), blood vessel (BV), and smooth muscle (SM)	NS	Surgical excision	No recurrence at 5 years
Fitzpatrick et al [11] (1990)	2	Male (77)	Lipoma vs cyst	NS	NS	Asymptomatic	NS	AT, BV, SM, and pseudocapsule (PSC)	NS	Surgical excision	No recurrence
Fitzpatrick et al [11] (1990)	3	Male (63)	Giant cell tumor of tendon sheath vs mucoid cyst	0.5	Toe	Asymptomatic	NS	AT, BV, SM, and PSC	NS	Surgical excision	No recurrence
Fitzpatrick et al [11] (1990)	4	Male (50)	NS	NS	Head	Asymptomatic	NS	AT, BV, SM, and PSC	NS	Surgical excision	No recurrence
Fitzpatrick et al [11] (1990)	5	Female (59)	NS	NS	Elbow	Asymptomatic	NS	AT, BV, SM, and PSC	NS	Surgical excision	No recurrence
Fitzpatrick et al [11] (1990)	6	Male (52)	Lipoma	1	Hand	Asymptomatic	NS	AT, BV, SM, and PSC	NS	Surgical excision	No recurrence
Fitzpatrick et al [11] (1990)	7	Male (33)	Epidermal cyst	3	Toe	Asymptomatic	NS	AT, BV, SM, and PSC	NS	Surgical excision	No recurrence
Fitzpatrick et al [11] (1990)	8	Male (48)	Lipoma	0.16	NS	Asymptomatic	NS	AT, BV, SM, and PSC	NS	Surgical excision	No recurrence
Fitzpatrick et al [11] (1990)	9	Male (39)	Subcutáneous nodule	NS	NS	Asymptomatic	NS	AT, BV, SM, and PSC	NS	Surgical excision	No recurrence
Mehregan et al [12] (1992)	10	Male (49)	Epidermal cyst	NS	Right helix	NS	NS	AT, BV, SM, and PSC	Negative	Surgical excision	No recurrence
Rodríguez-Fernandez et al [13] (1993)	11	Male (58)	NS	15	Elbow	Asymptomatic	4×3	AT, BV, SM, PSC, and atypia	NS	Surgical excision	No recurrence at 15 months
Ortíz-Rey et al [14] (1996)	12	Male (63)	Angioma	NS	Right preauricular area	Asymptomatic	1.5	AT, BV, SM, and PSC	Negative	Surgical excision	No recurrence at 11 months
Lee et al [15] (1996)	13	Male (32)	Lipoma vs epidermal cyst	5	Left earlobe	Asymptomatic	1.5×1.2	AT, BV, and SM	NS	Surgical excision	No recurrence
Val-Bernal et al [16] (1996)	14	Male (49)	Vascular tumor vs lipoma vs cyst	5	Right earlobe	NS	2.5×2	AT, BV, SM, and PSC	Negative	Surgical excision	No recurrence
Büyükbabani et al [17] (1998)	15	Male (38)	NS	10	Right retroauricular area	Asymptomatic	2.5×2.5	AT, BV, SM, and PSC	Negative	Surgical excision	2 previous recurrences in the same site following incomplete surgical excision
Büyükbabani et al [17] (1998)	16	Male (36)	NS	1.5	Nose	Asymptomatic	1.5×1.5	AT, BV, SM, and PSC	Negative	Surgical excision	NS
Castro-Forns et al [18] (1998)	17	Male (47)	NS	0.5	Nose	NS	1×0.7	AT, BV, and SM	NS	Surgical excision	NS
Castro-Forns et al [18] (1998)	18	Female (65)	NS	NS	Lumbar	NS	5	AT, BV, and SM	NS	Surgical excision	NS
Obata et al [19] (2001)	19	Female (54)	Lipoma vs cavernous angioma vs arteriovenous hemangioma	5	Nose	Asymptomatic	NS	AT, BV, SM, and PSC	NS	Surgical excision	No recurrence at 1 year
Tsuruta et al [20] (2004)	20	Male (75)	Lipoma	10	Left lateral nose over nasal cartilage	NS	NS	AT, BV, SM, and PSC	NS	Surgical excision	No recurrence at 7 years
Carlos de la Torre et. al [21] (2004)	21	Female (35)	NS	10	Palm - hypothenar region	Painful at touch	1.5	AT, BV, and SM	NS	Surgical excision	NS
Beer et al [22] (2005)	22	Male (43)	NS	0.5	Left ear	Asymptomatic	0.4	AT, BV, and SM	Negative	Surgical excision	No recurrence at 23 months
Beer et al [22] (2005)	23	Male (56)	NS	NS	Chin	Fluctuation in size with time	0.6	AT, BV, and SM	Negative	Surgical excision	No recurrence at 23 months
Beer et al [22] (2005)	24	Female (44)	Cyst	0.25	Left helix	Fluctuation in size and warm, ticklish sensation	0.5 cm	AT, BV, and SM	Negative	Surgical excision	No recurrence at 23 months
Debloom et al [23] (2006)	25	Female (50)	Epidermoid cyst vs lipoma vs leiomyoma	5	Left anterior proximal thigh	Asymptomatic	2.8×2	AT, BV, SM, and PSC	Negative	Surgical excision	NS
Makino et al [24] (2006)	26	Female (16)	Vascular tumor	NS	Buttock	NS	2.5×1.5	AT, BV, SM, and poorly circumscribed	Negative	Surgical excision	No recurrence at 2 years
Hyo Chan Jang et al [25] (2006)	27	Male (57)	Epidermal cyst	4	Left retroauricular area	Asymptomatic	2×1.5	AT, BV, SM, and PSC	Negative	Surgical excision	NS
Singh et al [26] (2009)	28	Male (45)	NS	NS	Chin	Asymptomatic	1	AT, BV, and SM	NS	Surgical excision	NS
Sánchez-Estella et al [27] (2009)	29	Female (58)	Angioma	5	Left retroauricular area	Change in size according to the ambient temperature	1.5	AT, BV, SM, and PSC	Negative	Surgical excision	No recurrence at 26 months
Sánchez-Estella et al [27] (2009)	30	Female (52)	Angiomyolipoma	2	Left retroauricular area	Change in size according to the ambient temperature	1	AT, BV, SM, and PSC	Negative	Surgical excision	No recurrence at 5 months
Shin et al [28] (2009)	31	Female (26)	Mucoid cyst	NS	Right helix	Asymptomatic	1×0.9	AT, BV, and SM	Negative	Surgical excision	No recurrence at 3 months
Mikoshiba et al [29] (2012)	32	Male (37)	Lipoma vs epidermal cyst	NS	Right earlobe	NS	1.7×1.6	AT, BV, and SM	Negative	Surgical excision	NS
Ammanagi, et al [30] (2012)	33	Female (3)	NS	NS	Anterior abdominal wall, below the umbilicus	NS	2.5	AT, BV, SM, and PSC	NS	Surgical excision	NS
Tchernev et al [31] (2014)	34	Female (66)	NS	NS	Right helix	NS	NS	AT, BV, and SM	NS	Surgical excision	No recurrence at 4 weeks follow up
Shim et al [32] (2014)	35	Male (45)	NS	NS	Right forehead	Asymptomatic	2×1.9	AT, BV, and SM	Negative	Surgical excision	No recurrence at 12-month follow-up
Han et al [33] (2014)	36	Male (36)	Vascular tumor	NS	Right nasal alar base	Asymptomatic	1×1	AT, BV, and SM	Negative	Surgical excision	NS
Yasar et al [34] (2014)	37	Male (67)	NS	10	Right earlobe	NS	2×2	AT, BV, and SM	NS	Surgical excision	No recurrence at 2 years
Carrau et al [35] (2015)	38	Male (13)	Neurofibroma	NS	First web space of the left foot	Asymptomatic	3.6×2.5	AT, BV, and SM	Negative	Surgical excision	NS
Kim et al [36] (2017)	39	Male (60)	NS	3	Glabella	Asymptomatic	2.3×1.7	AT, BV, SM, and PSC	Negative	Surgical excision	No recurrence at the 15 months
Mannan et al [37] (2019)	40	Male (36)	NS	NS	Right earlobe	NS	1.8×1.5	AT, BV, and SM	Negative	Surgical excision	NS
Araujo et al [38] (2020)	41	Male (32)	Epidermal cyst vs lipoma	4	Right earlobe	NS	1.3×1	AT, BV, and SM	Negative	Surgical excision	No recurrence at 44 months
Araujo et al [38] (2020)	42	Male (52)	Epidermal cyst vs lipoma	6	Right earlobe	NS	2.6×2.2	AT, BV, and SM	Negative	Surgical excision	No recurrence at 28 months
Oluwapelumi et al [39] (2020)	43	Female (11)	NS	11	Tip of nose	Recurrent mucus discharge, nasal blockage, and snoring	4×2	AT, BV (some cystically dilated), and SM	Negative	Surgical excision	No recurrence at 3 months
This study (2022)	44	Male (35)	Keloid scar vs skin adnexa tumor	Around 5 years	Right retroauricular area	Painful at touch	1.3×0.6	AT, BV, SM, and PSC	Negative	Surgical excision	No recurrence at 1 month

### Classic AML

#### Overview

Classic AML is a benign mesenchymal tumor composed of thick-walled blood vessels, mature adipose tissue, and bundles of smooth muscle cells in variable proportions. It was previously described as a hamartomatous lesion; however, molecular studies revealed its clonality and neoplastic nature [2,8,40]. It presents almost exclusively in the kidney (99.7%) [2,8,41,42] and is therefore further classified as renal or extrarenal. Extrarenal AMLs (0.3%) have been reported in the liver (most common extrarenal AML) [43-51], spleen [52], retroperitoneum [53], nasal cavity [54], oral cavity [55,56], heart [57,58], colon [59], lung [60], vagina [61,62], ovary [63], fallopian tubes [64], mediastinum [65], spermatic cord [66], penis [67], bone [68], and skin [69].

#### Etiology and Pathogenesis

Classic AML belongs to the perivascular epithelioid cell tumor (PEComa) family, which also includes lymphangioleiomyomatosis [40,70-73], clear cell “sugar” tumor [40,74-79], clear cell myomelanocytic tumor of the falciform ligament or ligamentum teres [80,81], abdominopelvic sarcoma of PECs [3-7], and cutaneous PEComa [82-85]. Classic AML is the most common PEComa [40].

Although all these tumors have distinct histologic features, they all originate from perivascular epithelioid cells, which have the peculiarity of coexpressing both melanocytic and myogenic markers. Therefore, these tumors probably originate from a cell with myomelanocytic differentiation, although no normal counterpart for this cell has been described [40,86].

The majority of classic AMLs are sporadic (80%). In comparison, up to 20% of them are associated with TSC [87,88]—a rare, autosomal dominant, multisystemic syndrome characterized by cutaneous abnormalities such as facial angiofibromas, ash-leaf macules, and shagreen patches—and diverse tumors, including classic AML (80% of patients with TSC) [2,40], subependymal giant cell tumor, cardiac rhabdomyoma, and lymphangioleiomyomatosis (LAM) [8,89]. Biallelic mutations in *TSC1* (~25%, hamartin in 9q34) and *TSC2* (~75%, tuberin in 16p13.3) [8,40,90-92] via point mutations, deletions, missense mutations, or copy neutral loss of heterozygosity [88,93] cause mTOR hyperactivation and consequently stimulate cell growth. Sporadic AML has also been associated with *TSC2* mutations [8,40,93]. TSC-associated classic AML tends to be bilateral and multifocal, while sporadic AML cases are isolated and unilateral [3,5,41].

Classic AML can also be associated with adult polycystic kidney disease, neurofibromatosis type 1 (NF1), and von Hippel-Lindau syndrome [32].

#### Epidemiology

Classic AML accounts for less than 1% of renal tumors; however, it is the most common renal mesenchymal tumor [8,87]. Sporadic classic AML has a female predilection (4:1) and occurs in patients between the age of 40-60 years, whereas TSC-associated classic AML has no gender predominance and occurs in patients between the age of 30-40 years [2,8,40,94].

#### Clinical Features

Most classic renal AMLs are asymptomatic and incidentally detected through imaging, surgery, or autopsy [8]. However, more than 80% of those larger than 4 cm are associated with abdominal or flank pain, hematuria, nausea, vomiting, fever, mass palpation [2,8], and renal failure (on rare occasions) [87], or new-onset hypertension [8]. Half of the symptomatic cases develop spontaneous bleeding, which may result in massive retroperitoneal hemorrhage and hypovolemic shock [2,8,41,95,96]. Rupture and bleeding during pregnancy are well-recognized complications [97,98]. Hence, tumors larger than 4 cm warrant prompt surgical intervention.

#### Radiologic Findings

Classic renal AML is easily diagnosed with uncontrasted computed tomography (CT) or magnetic resonance imaging (MRI) because of its abundant fat tissue. In 2016, Song et al [99] established a radiologic classification of renal AML as being “fat-rich,” “fat-poor,” or “fat-invisible”; the latter can have overlapping radiologic features with renal cell carcinoma and may often require percutaneous biopsy for adequate diagnosis [99-102].

#### Macroscopic Features

Classic AML is a yellow-white, smoothly rounded tumor with well-circumscribed, nonencapsulated borders. Its appearance varies depending on the proportion of adipose, vascular, and muscular components present [2-8,41]. Tumor size is variable, with those of sporadic cases ranging 1-30 cm (median 9 cm), while those of TSC-associated cases are usually smaller and can be multiple [2,103].

#### Microscopic Features

Classic AML comprises the characteristic triad of thick-walled blood vessels devoid of lamina elastica, mature adipose tissue, and bundles of spindled or epithelioid smooth muscle cells [2-8,41,42,87]. Hemorrhage and necrosis are commonly detected [8].

There are several histologic variants, including microscopic AML (absent thick-walled blood vessels) [104,105], intraglomerular AML (epithelioid smooth muscle cells intermixed with a few adipocytes in capillary tufts) [106,107], AML with epithelial cyst (cysts, “cambium-like” stromal cells, solid smooth muscle predominant areas, prominent lymphovascular network, and rare adipose tissue) [108,109], lymphangiomatosis of the renal sinus (plaque-like mass in the renal pelvis) [110], sclerosing AML (cords and trabeculae of bland epithelioid cells in abundant sclerotic stroma) [111], and epithelioid AML (EAML) [40,87,104,112]; the latter has distinct implications that require further description.

EAMLs (5%-7% of classic AML) require more than 80% of epithelioid morphology [8,40,104], consequently reducing the proportion of blood vessels and adipose tissue. It has varying degrees of nuclear atypia and may contain multinucleated giant cells. This rare subtype is potentially malignant and may exhibit aggressive behavior such as recurrence, invasion into the inferior vena cava, and metastasis (to the lungs, bone, and liver) [8]. Brimo et al [113] established a model to predict malignant and aggressive clinical behavior in EAMLs when finding 3 or more of the following: ≥70% of atypical epithelioid cells, ≥2 mitotic figures per 10 high-power fields, atypical mitotic figures, and necrosis. Hence, EAMLs must be monitored closely.

#### Immunohistochemistry

Classic AML is typically positive for melanocytic markers (95%) such as HMB-45 (expressed in a patchy pattern), Melan-A, Micropthalmia transcription factor, and Tyrosinase [2,8,40,114]. Smooth muscle cells are also immunoreactive to myogenic markers such as SMA, Calponin, and Desmin [8]. Other positive markers include cathepsin K [2,8,40] and, less frequently, CD117, CD68, S-100, estrogen receptor, and progesterone receptor (more common in the epithelioid variant) [2,8,40,115-117].

#### Treatment

Surgical management is recommended in AMLs with a tumor size greater than 1 cm, symptomatic patients, or those with a high risk of tumor bleeding or rupture. Some tumors have been treated with embolization. In some cases, medical therapy with mTORC1 inhibitors, such as sirolimus, has shown a positive clinical response and prevented renal failure [40,101,118,119].

Asymptomatic patients with AMLs smaller than 1 cm and those with significant comorbidities with AMLs smaller than 3 cm should be followed up periodically with CT or MRI [101].

#### Prognosis

Recurrence in classic AML is rare; however, approximately 25% of cases of EAML with atypia can recur, metastasize, and cause cancer-related death [8,114]. In a series of 41 cases of pure (monotypic) epithelioid cell PEComa neoplasms, Nese et al [120] observed recurrence in 17%, metastasis in 49%, and cancer-related death in 33% of cases.

### Cutaneous AML

#### Overview

Cutaneous AML is demographically, clinically, and immunohistochemically distinct from its classic counterpart (Tables 1 and 2). Cutaneous AML, previously termed cutaneous angiolipoleiomyoma [11,24,34], is a rare, benign tumor with varying proportions of thick-walled blood vessels, adipose tissue, and smooth muscle cell bundles.

**Table 2:** Cutaneous versus classic angiomyolipoma.

	Classic angiomyolipoma	Cutaneous angiomyolipoma
Demographic data	Predominant in females	Predominant in males
Etiopathogenesis	Perivascular epithelioid cell tumor; 20% associated with tuberous sclerosis complex	One case associated with neurofibromatosis type 1
Clinical	Almost exclusively in the kidney; median size 9 cm	More frequent in the ear; median size 1.5 cm
Morphology	Epithelioid angiomyolipoma with varying atypia, mitosis, and necrosis	No atypia, mitosis, or necrosis
Immunohistochemistry	Positive melanocytic markers	Negative melanocytic markers
Prognosis	Epithelioid angiomyolipoma can recur, metastasize, and cause cancer-related death	Resolution following complete surgical excision

#### Epidemiology

Unlike its classic counterpart, cutaneous AML occurs predominantly in males (70%). The age range is wide (2-77 years), with a peak incidence between the age of 30-50 (median 48) years.

This tumor occurs predominantly in the head (76%) but has also presented in the limbs (22%) and abdomen (2%). Of the head tumors, the ear was the most frequent location in 62% of cases, followed by the nose in 19%, and, less frequently, in the forehead, chin, and eyelid (19%).

#### Clinical Features

Most patients are asymptomatic, presenting only with a visible or palpable nodular lesion with slow growth, ranging from 2 months to 40 years (median 5 years). Some patients experience tumor size fluctuation over time or that associated with environmental temperature changes (clinical manifestation of the vascular component of the tumor) [22,28], pain (probably associated with increased sensitivity due to location or trauma) [21], and obstructive symptoms related to specific sites (such as nasal cavity) and large tumor size [39].

In the majority of cases, cutaneous AMLs are clinically misdiagnosed. The most common clinical diagnoses are cystic lesions (35%, mainly epidermoid cysts), lipomas (28%), and benign vascular tumors (17%; Table 1), the latter two being consistent with the tumors’ components.

No cases of cutaneous AML have been associated with TSC to date. Only one case of AML in the skin in a patient with TSC has been reported [69]; however, this tumor had all the features of classic AML (including expression of melanocytic markers), which suggest classic AML with skin extension rather than a true cutaneous AML. A sole case of true cutaneous AML was reported in a patient with NF1 [35].

#### Radiologic Findings

Owing to its superficial location and easily accessible surgical approach, imaging studies are usually unnecessary for diagnosis. In the few cases reported, CT and MRI confirmed adipose and vascular components [33], similar to classic AMLs’ radiologic findings.

#### Macroscopic Features

Cutaneous AMLs are well-circumscribed, whitish-gray dermal tumors, measuring 0.4-5 (median 1.5) cm, generally smaller than their classic counterpart (median 9 cm).

#### Microscopic Features

Histologically, most cases are well-circumscribed, with an admixture of small to medium, thick-walled, muscular blood vessels (some dilated and containing thrombi), mature adipose tissue, and smooth muscle bundles in variable proportions, identical to classic AML.

Half of the cutaneous AMLs are surrounded by a fibrous pseudocapsule, probably as a stromal response to tumor growth. Some cases present epidermal changes such as atrophy or hyperplasia. Faint chronic inflammatory infiltrate was also present in some cases [16,22].

Unlike classic AML, there is no epithelioid variant in cutaneous AMLs; consequently, they do not display cellular atypia, necrosis, or mitosis. Only one case had pleomorphic and bizarre nuclear changes in the smooth muscle component [13]; however, the absence of epithelioid cells, mitotic activity, necrosis, and the prolonged clinical duration (15 years) support the degenerative nature of these findings, similar to those observed in ancient schwannomas [13,121].

#### Special Stains

When requested, Masson’s trichrome staining revealed smooth muscle cells in red and collagen fibers (present in the stroma and fibrous pseudocapsule) in blue. Elastic fiber staining shows an absent or defective lamina elastica in some vessels.

#### Immunohistochemistry

Cutaneous AML is characteristically positive for smooth muscle markers such as SMA, Calponin, and Desmin. However, unlike classic AML, all cutaneous AMLs are negative for melanocytic markers such as HMB-45, Melan-A, MART-1, and SOX-10. Other frequently positive markers include S-100, Factor VIII, CD31, CD34, and FLI1.

#### Treatment and Prognosis

Complete surgical excision is the diagnostic and therapeutic procedure indicated for cutaneous AML; these tumors are usually easily “shelled out” [11,12,23]. Cutaneous AMLs are always benign, do not progress, and only recur if excision is incomplete [17], highlighting the importance of complete removal with negative margins.

#### Differential Diagnosis

In the skin, some tumors are composed of one or more of the AML components. Angiolipoma is composed of mature fat cells and clusters of thin-walled capillaries and lacks smooth muscle bundles. Although angioleiomyoma is also characterized by thick-walled blood vessels (as in AML), its smooth muscle cells are arranged concentrically around blood vessels, and it lacks adipose tissue. Arteriovenous malformation is composed of large-caliber arteries, arterioles, capillaries, venules, and thick-walled veins; however, it lacks smooth muscle bundles and adipose tissue [1].

The most important differential diagnosis is classic AML in the skin [69] since they are histologically identical. The expression of melanocytic markers and distinct demographic/clinical features (previously described) are crucial for proper differentiation between these two entities.

### Conclusions

Owing to the rarity of cutaneous AML, it is currently not included in the 2018 WHO classification of skin tumors [1]. Moreover, the current information still associates these tumors as cutaneous presentations of the classic AMLs with some differences.

Our review strongly suggests that cutaneous and classic AMLs must be considered separate entities. In summary, the main differences reside in the following aspects:

- Clinical: predominantly in males, more frequent in or around the ear, and presenting exclusively as a solitary lesion.
- Etiopathogenesis: without any reported association with TSC.
- Morphology: lacking aggressive variants such as EAML, necrosis, and atypical mitoses.
- Immunohistochemistry: absent melanocytic markers.
- Prognosis: benign behavior with lack of recurrence following complete surgical excision.

The immunohistochemical findings discard PECs or any other cell with melanocytic differentiation as a possible origin for cutaneous AML; hence, unlike classic AML, this tumor does not belong to the PEComa family. It is reasonable to consider cutaneous AML as a true and pure “angio-myo-lipoma.”

Future updates of the WHO classification of skin tumors should consider including cutaneous AML as a separate entity. Finally, physicians should be aware of the possibility of a cutaneous AML when presented with a nodular mass in the ear, as appropriate treatment can provide patients with complete clinical resolution.

## Abbreviations

AML: angiomyolipoma
CT: computed tomography
EAML: epithelioid angiomyolipoma
MRI: magnetic resonance imaging
NF1: neurofibromatosis type 1
PEC: perivascular epithelioid cell
PEComa: perivascular epithelioid cell tumor
SMA: smooth muscle actin
TSC: tuberous sclerosis complex
WHO: World Health Organization
